# Empirical analysis of AS-level cooperation on the internet considering geopolitical characteristics

**DOI:** 10.1371/journal.pone.0347291

**Published:** 2026-04-20

**Authors:** Jiaqi Nie, Tao Jin, Zhenyu Wang, Chenbo Fu, Shanqing Yu, Qi Xuan, Zhongyuan Ruan

**Affiliations:** 1 Institute of Cyberspace Security, Zhejiang University of Technology, Hangzhou, Zhejiang, China; 2 Binjiang Institute of Artificial Intelligence, Zhejiang University of Technology, Hangzhou, Zhejiang, China; University College London - Bloomsbury Campus: University College London, UNITED KINGDOM OF GREAT BRITAIN AND NORTHERN IRELAND

## Abstract

The Internet is a complex socio-technical system intricately embedded within physical geography and political contexts. While previous studies have emphasized the technical aspects of its topological structure, the geopolitical dimension of the Internet has not been fully explored yet. In this paper, we integrate multiple sources of data — including AS relationships and their national or regional affiliations — to study the Internet at the Autonomous Systems (AS) level, with a particular focus on cooperation patterns among ASes. Our findings reveal that the global AS network exhibits moderate disassortative mixing and weak nestedness, suggesting a flattening trend. However, within many individual countries or regions, AS cooperation patterns display stronger disassortativity and greater nestedness than the global average, indicating a more notable hierarchical organization. We further propose a visualization method to track the changes of cooperation trends among countries/regions. Finally, through case studies of the Russia-Ukraine conflict and Pelosi’s Visit to China’s Taiwan Region, we show that international AS-level cooperation (particularly for the countries/regions involved) may undergo rapid and significant changes in response to geopolitical events.

## Introduction

The operations of modern society rely heavily on the Internet, which is one of the largest self-organizing systems and has been extensively studied through network frameworks [1–3]. In practice, researchers typically examine it at the Autonomous System (AS) level of granularity, where an AS represents a collection of computers and routers that share a unified routing policy [4,5]. In this context, the Internet can be viewed as a vast network, with nodes representing ASes and edges denoting the cooperative relationships between them. Notably, each AS is registered under a specific country or region, and their spatial distribution is highly uneven across regions, influenced by geopolitical policies, historical path dependencies, and economic inequalities [6–8]. This uneven distribution underscores that the Internet’s topology is deeply embedded in its geopolitical context.

In recent years, the geopolitical significance of the Internet has increasingly attracted attention. For example, some studies showed that analyzing the geographic belonging of ASes can reveal policy differences within countries/regions [9–11], as well as cross-border international relationships [12,13]. The hierarchical structure of AS ecosystems also reflects these geopolitical dimensions: Tier-1 ASes, typically controlled by entities in a few dominant countries, form the global backbone, while Tier-2 ASes often establish provider-customer relationships across borders, resulting in complex interdependencies [14].

Although existing research has advanced our understanding of the Internet’s topological characteristics (e.g., the k-core structure and the rich-club effect [15–17]), the geopolitical dimensions of AS-level cooperation remain insufficiently explored. In this study, we integrate multiple sources of data, including AS relationship data from January 2015 to December 2024 (separately for IPv4 and IPv6) and AS ownership data labeled by corresponding countries or regions, to investigate the cooperation patterns of the Internet at the AS level. We further examine the impact of geopolitical events on the cooperation patterns. Our study reveals a trend of hierarchical organization within individual countries/regions, and a trend of flattening at the global Internet level. Moreover, the cooperation among the involved countries/regions may change rapidly and noticeably in response to geopolitical events.

## Dataset

Our analysis requires constructing a network that integrates three key elements: (1) AS relationship topologies, (2) temporal network slices, and (3) country/region affiliation labels for AS nodes. This network was constructed using the following data sources:

- **AS relationships [18].** The Center for Applied Internet Data Analysis (CAIDA) has provided AS relationships datasets since 1998, inferred from BGP data using their proprietary algorithm. In this study, we focus on both IPv4 and IPv6 networks, utilizing 120 monthly snapshots for each network from January 2015 to December 2024. We model the AS network as an unweighted and undirected graph *G* = (*V*, *E*), where *V* denotes the set of ASes and *E* represents the inferred inter-AS relationships.
- **Codes for Countries and Their Subdivisions [19].** We refer to the ISO 3166 two-letter country codes and names, which are internationally recognized and based on authoritative sources from the United Nations, including the Terminology Bulletin Country Names and the Country and Region Codes for Statistical Use.
- **Country codes of ASes [20].** Regional Internet Registries (RIRs) have adopted a standardized file format to report the current state of Internet resource allocations and assignments. This format covers IPv4 address ranges, IPv6 address ranges, and AS numbers (ASN). The file provides detailed information on address space allocations and assignments, including the country/region where the resources were initially allocated or assigned.

## Results

### Overall cooperation pattern

**Fig 1:**
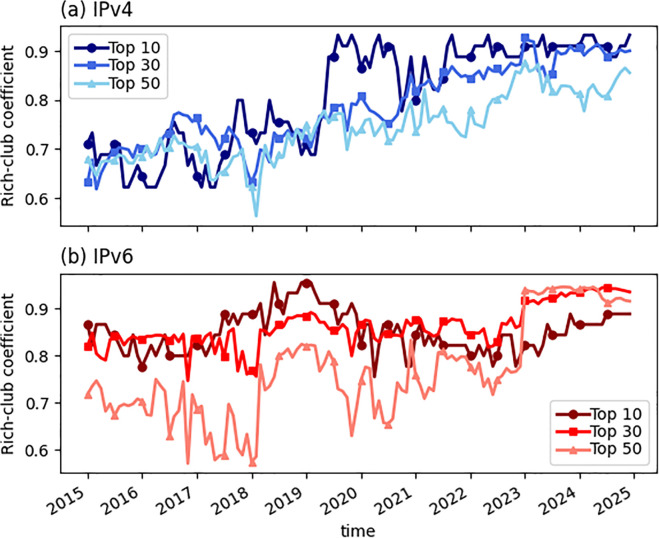
Rich-club coefficients in IPv4 and IPv6 Networks. (a) and (b) show the rich-club coefficients for the top-*n* (*n* = 10,30,50) highest-degree nodes in the IPv4 and IPv6 networks, respectively, from 2015 to 2024.

Previous studies have revealed some fundamental properties of the Internet. For instance, the AS networks (including both IPv4 and IPv6) have been continuously expanding over time. As of December 2024, the IPv4 network contains 77,495 nodes and 501,473 links, while the IPv6 network includes 13,994 nodes and 154,537 links. The degree distributions of both networks exhibit power-law–like behavior, indicating a highly heterogeneous structure. Moreover, the Internet exhibits high values of the rich-club coefficient (defined as the ratio of the actual number of links to the maximum possible number of links among a group of high-degree nodes [15,21,22]), indicating that the high-degree ASes are strongly interconnected [23]. As shown in Fig 1, the rich-club coefficients for the top-*n* (*n* = 10,30,50) highest-degree nodes in both IPv4 and IPv6 networks maintain consistently high values from 2015 to 2024. This structural property is crucial for the Internet’s routing efficiency and overall robustness.

It is worth noting that the rich-club coefficient is an absolute measure of the interconnectivity density among rich nodes [23], which differs from the closely related concept of rich-club ordering proposed by Colizza et al [24]. Rich-club ordering emphasizes normalizing the rich-club coefficient against that of a randomized null model. Under such a framework, the internet appears to exhibit only a weak rich-club phenomenon. However, this approach (as well as several of its modified versions [6]) is not well suited for meaningful comparisons between different real networks. For example, strong rich-club ordering in one network does not imply that its rich nodes are more densely interconnected than those in another network with weak rich-club ordering, just as a primary school student who is tall relative to his class may still be shorter than a basketball player who is short relative to his team [23].

**Fig 2:**
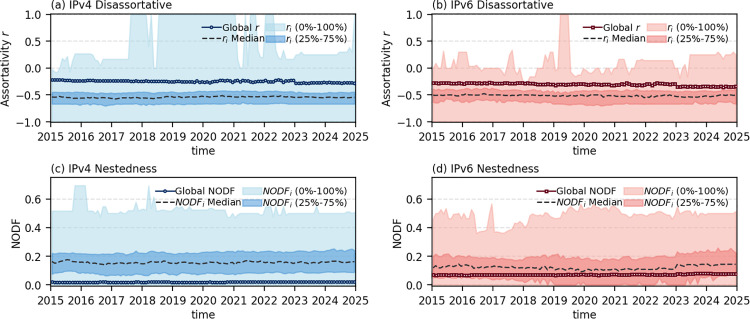
Cooperation pattern within individual countries/regions. (a) and (b) show the median assortativity coefficients (*r*) and the corresponding full (0% − 100%) and interquartile (25% − 75%) ranges for country/region-level sub-networks (consisting of ASes with identical country/region labels) in IPv4 and IPv6 networks at each time snapshot. (c) and (d) present the median *NODF* values and corresponding percentile ranges for the same set of sub-networks in IPv4 and IPv6 networks at each time snapshot, respectively. Circles or squares in the panels represent the assortativity or *NODF* value of the global network without country/region partitioning.

Here we focus on two well-studied concepts in network science, i.e., assortativity and nestedness, to illustrate the overall cooperation patterns among ASes in the Internet. Assortativity is a metric used to measure the connection tendencies between node degrees, which can be quantified using Pearson’s correlation coefficient *r* between the degrees of two connected nodes [25]. The values of *r* range from −1 to 1. For *r* < 0, the network is disassortative, meaning that high-degree nodes tend to connect with low-degree nodes. In contrast, *r* > 0 indicates assortative mixing, where nodes preferentially link to others with similar degrees. Previous studies have shown that AS networks typically exhibit disassortative mixing, where small transit providers prefer to link with larger transit provider ASes, thereby facilitating more efficient information routing. To confirm this, we plot the assortativity coefficient *r* as a function of time for both IPv4 and IPv6 networks, and observe that both networks display moderate disassortative mixing throughout the entire period (see the circles and squares in Fig 2 (a) and (b), respectively).

Nestedness is another important network property that reflects the extent to which a node’s neighbors are subset of the neighborhoods of higher-degree nodes [26]. A higher level of nestedness implies a more pronounced hierarchical organization, where low-degree nodes rely heavily on high-degree nodes and have few links among themselves. In the context of AS networks, it means that routes are advertised in a strictly top-down manner, leaving stub ASes with almost no alternative paths. Nestedness has been extensively studied in both bipartite and unipartite networks [27,28], and prior work has confirmed its presence in AS-country bipartite networks [29]. Here, we focus on investigating nestedness in unipartite AS networks. We use the Nested Overlap and Decreasing Fill (*NODF*) to quantify nestedness, where higher values indicate stronger nestedness [30]. As shown by the circles and squares in Fig 2 (c) and (d), we see that both IPv4 and IPv6 networks show a stable weak nestedness over time (particularly for the IPv4 network, the *NODF* is close to 0). It is worth noting that previous studies have revealed a strong correlation between nestedness and disassortativity [31]. However, in the case of AS networks, such correlation is relatively weak.

To summarize, the AS networks exhibit moderate disassortativity and weak nestedness throughout the observation period. This indicates that the AS ecosystem is relatively flat, where some high-degree ASes play a central role in maintaining global connectivity, while the overall structure is far from hierarchical.

### Cooperation within individual countries/regions

We next shift our focus to the local structures of the global network by considering individual countries and regions. We calculate the values of *NODF*_*i*_ and *r*_*i*_ for each subnetwork *i*. Fig 2 (a) and (b) show the distribution of *r*_*i*_ over time in the IPv4 and IPv6 networks. It can be observed that different countries and regions exhibit significant variation, reflecting the underlying diversity in policies, markets, and infrastructure. In general, although a few countries/regions exhibit strong assortativity, the majority are disassortative, with coefficient *r*_*i*_ lower than the global network (highlighted by the circles in the figure). Correspondingly, the distributions of *NODF*_*i*_ for IPv4 and IPv6 networks are presented in Fig 2 (c) and (d). Most values of *NODF*_*i*_ are higher than the global *NODF*, indicating a more pronounced nested structure at the local level. These results suggest that while the global Internet is flattening and lacks clear nestedness, many individual countries and regions exhibit local centralization dependencies and hierarchical structures. Within these areas, a few backbone ASes typically provide access services to other AS nodes.

### Cooperation among countries/regions

To further investigate how ASes cooperate both inside and outside countries/regions, we treat each country/region as a module and apply modularity *Q* [32] to quantify the cooperation patterns. The value of *Q* ranges from −0.5 to 1, with higher values indicating denser connections within modules and sparser connections between them. In this context, we refer to edges that connect nodes within the same country or region as intra-edges (intra-country/region cooperation), while edges connecting between different countries/regions as inter-edges (international cooperation). It is important to note that AS networks have been increasing in edge density over time [6], which may significantly influence modularity measurements.

**Fig 3:**
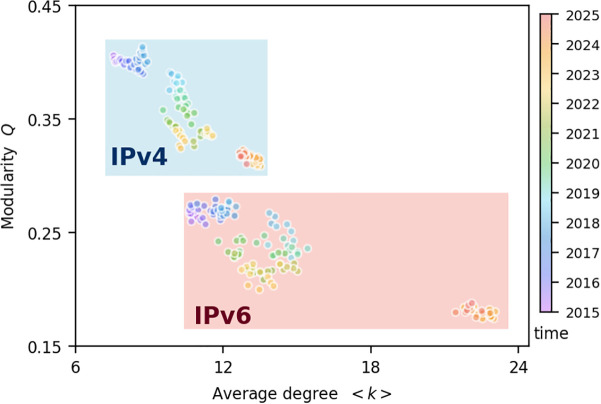
Cooperation among countries/regions. Relationship between modularity *Q* and average degree ⟨k⟩ in IPv4 and IPv6 networks. Each dot corresponds to a snapshot of the network, with its color indicating the corresponding time. Results for IPv4 and IPv6 networks are highlighted with blue and red shading, respectively.

Fig 3 illustrates the relationship between the modularity *Q* and average degree ⟨k⟩ for both IPv4 and IPv6 networks over time. Nodes in different colors represent distinct time snapshots. Notably, as time progresses, the average degree increases while modularity decreases. Although this trend is consistent across both networks, the IPv6 network exhibits a higher ⟨k⟩ and a lower *Q* (see the red shaded area in the figure). The increase in the number of edges can influence modularity in two ways: if edges are added within modules, *Q* tends to increase; whereas if they are added between modules, *Q* tends to decrease. In other words, these two types of edges have opposing effects on modularity.

**Fig 4:**
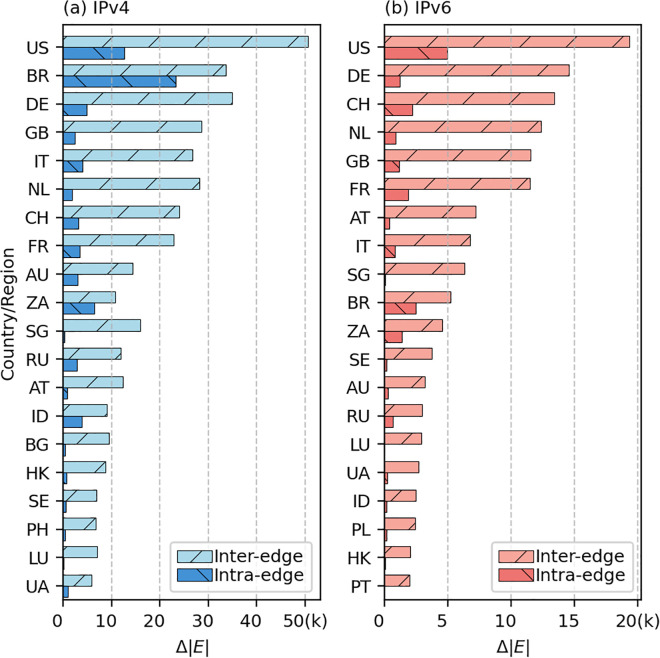
Number of edges in TOP20 countries/regions. Variations in the number of intra- and inter-edges for countries/regions over the entire 120-month period in **(a)** IPv4 and **(b)** IPv6 networks. Countries/regions are sorted by their contributions to the total number of edges in the respective networks, and the top 20 are presented.

The decline in *Q* over time indicates that the number of inter-module edges plays a dominant role in shaping the network structure, leading to the blurring of boundaries between countries/regions. To confirm this, we select 20 representative countries/regions in both the IPv4 and Ipv6 networks (these countries/regions contribute 80% of the changes in the total number of edges in their respective networks) and present the changes in their intra- and inter-edge numbers over the entire 120-month period. The results are shown in Fig 4. This phenomenon reflects the ongoing globalization of the Internet. Countries/regions with many ASes, such as the United States, typically have more Data Centers and Internet Exchange Points (IXPs) [33]. In contrast, many less developed countries and regions rely on resource-rich counterparts as customers to meet their needs.

### Visualization of cooperation tendency

The analysis above reveals an increasing trend in both the total number of edges and the number of inter-edges among countries/regions over time. To intuitively demonstrate these changes, we propose a visualization method to track variations in cooperative tendencies across different countries/regions over the past decade. Here, we focus on two key aspects: (1) The growth in the number of edges involving each country/region, and (2) the dynamic changes in cooperative tendencies (i.e., whether there is a strong preference for internal or external cooperation). In the experiment, we compute both the total number of edges and the proportion of inter-edges for each country/region at every time snapshot. The inter-edge proportion for a given country/region is defined as the number of inter-edges it participates in divided by its total number of edges. Given that each country/region has data across 120 time points, taking a country/region *C*_1_ in IPv4 as an example, we obtain a sequence of the number of edges: $\left[|E_{t=1}^{C_{1}}|,|E_{t=2}^{C_{1}}|,...,|E_{t=120}^{C_{1}}|\right]$, as well as a corresponding sequence for the inter-edge proportions: $\left[ratio_{t=1}^{C_{1}},ratio_{t=2}^{C_{1}},...,ratio_{t=120}^{C_{1}}\right]$ (similarly for IPv6). These two sequences are essential for tracking detailed changes in both network scale and cooperative tendencies for each country/region.

**Fig 5:**
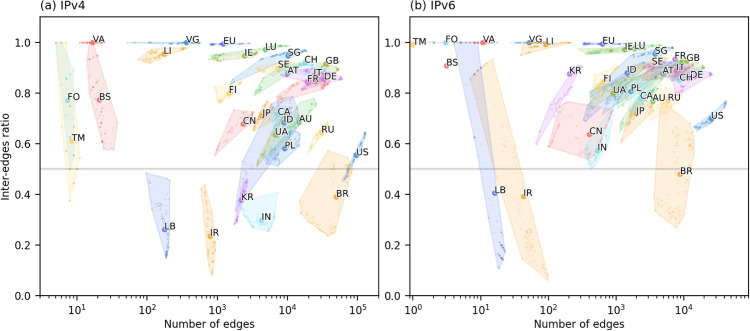
Visualization of cooperation tendency. Visualization of international cooperation tendencies for countries/regions in **(a)** IPv4 and **(b)** IPv6 networks. Each shaded area with labels reflects the changes in the number of total edges and the proportion of inter-edges of that country/region over the past decade. Dots within each shadow indicate the specific values at each time snapshot.

We then transform these two sequences into graphical representations. For each time point, the data pair ($|{E^{C}}_{t}|,{ratio^{C}}_{t}$) from the two sequences is treated as a point in a two-dimensional plane, forming a series of points over time. Next, we apply a convex hull algorithm (a standard geometric abstraction widely used in spatial analysis and available in SciPy through scipy.spatial.ConvexHull function [34]) to highlight the area covered by these points with shading. Using this method, we selected several representative countries/regions, as illustrated in Fig 5.

If the network size has grown significantly over the past decade, the number of edges will vary considerably, resulting in a wide horizontal spread of the corresponding shading. On the other hand, if the proportion of inter-edges exceeds 0.5, it suggests a stronger tendency for the country/region to cooperate with external entities. As shown in Fig 5, the majority of countries/regions exhibit inter-edge proportions above 0.5.

For the IPv4 network as shown in Fig 5 (a), three primary patterns of change in cooperation tendencies can be identified: (1) Increasing external cooperation. This is represented by bands sloping upward to the right at different angles, indicating a tendency to cooperate with external entities. Examples include US, CN, RU, AT, and AU. This pattern suggests that these countries/regions have expanded their network size while increasingly establishing connections with external entities. (2) Increasing internal cooperation. This is characterized by bands sloping downwards to the right, hinting a tendency to cooperate internally. Examples include IR, BS, FO, TM, and LB. This pattern may be attributed to geopolitical complexity, economic and industrial characteristics. (3) Consistently high external cooperation. This is represented by nearly horizontal bands at high inter-edge ratios (≥ 0.9). Examples include EU, LU, IE, and VG.

For the IPv6 network as shown in Fig 5 (b), the shapes for most countries/regions are similar to those in the IPv4, though some differences can be observed. Due to the later development of IPv6, the number of edges is relatively small. The cooperative tendencies of the same country/region in the IPv4 and IPv6 networks can differ significantly. For example, KR and IR both have an inter-edge ratio greater than 0.5 in the IPv6 network but less than 0.5 in the IPv4. Moreover, CN exhibits significant shift both in its inter-edge ratio and edge numbers. The distribution of the data points reveals that there is a decline followed by a rebound in the inter-edge ratio, which may be related to domestic policies promoting large-scale deployment of hardware infrastructure to support the development of the IPv6 [35].

### Impact of geopolitical events

Finally, it is worth emphasizing that AS-level cooperation is highly sensitive to geopolitical events. To illustrate this, we focus on two typical cases: the Russia-Ukraine conflict and Pelosi’s visit to China’s Taiwan region. For the first event, we analyze changes of the top five cooperation partners of Ukraine (UA) (ranked by the number of shared edges) before and after the onset of the conflict. For the latter, we examine the shifts in cooperation patterns of Taiwan (TW) surrounding the visit.

**Fig 6:**
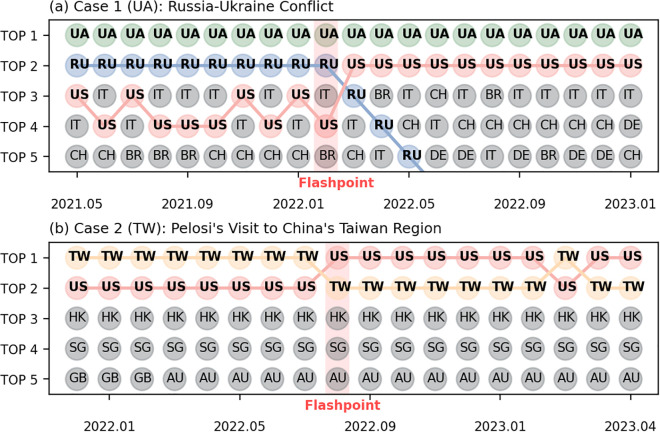
Visualization of Geopolitical Events Impact. Case studies on the impact of geopolitical events on international cooperation for the countries/regions involved. (a) and (b) show how the top five countries/regions with which Ukraine and the Taiwan region of China most frequently cooperated in terms of AS-level connections changed before and after the respective events. The month in which each event occured is highlighted with red shading.

The Russia-Ukraine conflict, which began in February 2022, has led to a sharp deterioration in relations between the two countries. As shown in Fig 6 (a), before the conflict, RU was the closest cooperative partner of UA, largely due to their geographical proximity. However, after the flashpoint, the rank of RU began to decline month by month, and the US replaced position of RU, which is related to the network support provided by the US at that time [36]. Another notable event was the visit of U.S. politician Nancy Pelosi to Taiwan. From Fig 6 (b), it can be seen that TW and the US already maintained a high level of cooperation prior to the visit. After the event, a further increase in cooperation can be observed, which may reflect shifts in political or economic engagement [37]. In summary, geopolitical events have a significant impact on the internet cooperation network, as evidenced by rapid shifts in cooperation among the involved countries and regions.

## Conclusion

In conclusion, we have investigated the Internet at the AS level across multiple granularities, ranging from individual AS nodes to countries and regions. Our findings reveal that, at the global level, AS networks exhibit disassortative mixing and weak nestedness, reflecting the flattening nature of the Internet. However, when focusing on local structures, we observed significant diversity across countries and regions. Many of them display stronger disassortative and greater nestedness compared to the global average, suggesting localized centralization and hierarchical organization. We further explored inter-country/region cooperation by treating each country/region as a module and observed a gradual decrease in network modularity. This trend indicates increasing cross-border cooperation over time. To capture these dynamics, we introduced a visualization method to track the changes in cooperation patterns for specific countries/regions. Finally, we examined the influence of geopolitical events and demonstrated that Internet cooperation responds rapidly and noticeably to such events. We hope that this study may contribute to a deeper understanding of Internet structure and dynamics, and inspire further research on the interplay between Internet topology and geopolitical influences.

## Supporting information

S1 File Supporting Information. (ZIP)
