# Supplementary material for: Empirical analysis of AS-level cooperation on the internet considering geopolitical characteristics
Source: PLoS One. 2026 Apr 20;21(4):e0347291. doi: 10.1371/journal.pone.0347291 (PMC13094963; doi:10.1371/journal.pone.0347291)
Supplement: S1 File — (ZIP) [file pone.0347291.s001.zip › Supporting Information.pdf]

Assortativity Coefficient The assortativity coefficient r is given by

$$r = \frac{\sum_{kk'} kk'(e_{kk'} - q_k q'_k)}{\sigma_q^2} \tag{1}$$

where $e_{kk'}$ is the fraction of edges connecting nodes of degree k and k', q_k is the distribution of the remaining degree of a node reached by following a randomly chosen edge, and σ_q^2 is the variance of the distribution q.

Nestedness Metric The Overlap and Decreasing Fill (*NODF*) was first defined on bipartite networks. Consider an incidence matrix with N rows and M columns. The row-*NODF* $\mathcal{N}^R$ is defined as

$$\mathcal{N}^R = \sum_{(i,j)} \frac{O_{ij}}{k_j} \Theta(k_i - k_j) \tag{2}$$

where (i, j) is a pair of row nodes, O_{ij} is the number of common neighbors between nodes i and j, k_i and k_j represent their respective degrees, and Θ denotes the Heaviside function: $\Theta(x) = 1$ if $x > 0$, $\Theta(x) = 0$ if $x \leq 0$.

Similarly, the column-*NODF* $\mathcal{N}^C$ is defined as

$$\mathcal{N}^C = \sum_{(\alpha,\beta)} \frac{O_{\alpha\beta}}{k_\beta} \Theta(k_\alpha - k_\beta) \tag{3}$$

Finally, the total *NODF* η is given by

$$\eta = \frac{\mathcal{N}^R + \mathcal{N}^C}{\frac{N(N-1)}{2} + \frac{M(M-1)}{2}} \tag{4}$$

In the case of unipartite graphs, where the adjacency matrix is square ($N = M$), the row-*NODF* and column-*NODF* are equivalent ($\mathcal{N}^R = \mathcal{N}^C$).

Modularity The modularity Q is given by

$$Q = \frac{1}{2|E|} \sum_{ij} \left(A_{ij} - \frac{k_i k_j}{2|E|} \right) \delta(c_i, c_j) \tag{5}$$

where A_{ij} is the element of the adjacency matrix, k_i and k_j are the degrees of nodes i and j, $|E|$ is the total number of edges in the network, and $\delta(c_i, c_j)$ is an indicator function that is 1 if nodes i and j belong to the same community and 0 otherwise.

Countries/Regions List The countries/regions involved in Figs. 4-5 include: the United States of America (US), Brazil (BR), Germany (DE), the United Kingdom of Great Britain and Northern Ireland (GB), Italy (IT), the Kingdom of the Netherlands (NL), Switzerland (CH), France (FR), Australia (AU), South Africa (ZA), Singapore (SG), the Russian Federation (RU), Austria (AT), Indonesia (ID), Bulgaria (BG), Hong Kong (HK), Sweden (SE), the Philippines (PH), Luxembourg (LU), Ukraine (UA), China (CN), India (IN), Islamic Republic of Iran (IR), the Bahamas (BS), the Faroe Islands (FO), Turkmenistan (TM), Lebanon (LB), the Republic of Korea (KR), Japan (JP), Liechtenstein (LI), the Holy See (VA), Canada (CA), Poland (PL), Portugal (PT), Ireland (IE), the British Virgin Islands (VG), Taiwan (Province of China) (TW), Finland (FI), and European Union (EU). It should be clarified that the label “EU” does not represent all EU countries. EU-labeled ASes often belong to organizations registered in Europe without specifying a particular country.
